# Pearls and pitfalls in comprehensive critical care echocardiography

**DOI:** 10.1186/s13054-017-1866-z

**Published:** 2017-11-17

**Authors:** Sam Orde, Michel Slama, Andrew Hilton, Konstantin Yastrebov, Anthony McLean

**Affiliations:** 1 Intensive Care Unit, Nepean Hospital, Kingswood, Sydney, NSW Australia; 2 Medical Intensive Care Unit, Amiens University Hospital, Amiens, France; 3 Intensive Care Unit, Austin Hospital, Heidelberg, Melbourne, VIC Australia; 4 Intensive Care Unit, St George Hospital, Kogarah, Sydney, NSW Australia

**Keywords:** Critical care, Intensive care, Echocardiography, Doppler, Advanced

## Abstract

Critical care echocardiography is developing rapidly with an increasing number of specialists now performing comprehensive studies using Doppler and other advanced techniques. However, this imaging can be challenging, interpretation is far from simple in the complex critically ill patient and mistakes can be easy to make. We aim to address clinically relevant areas where potential errors may occur and suggest methods to hopefully improve accuracy of imaging and interpretation.

## Background

Cardiac dysfunction is commonly associated with critical illness and often involves both ventricles. It can result from chronic underlying conditions (often unknown prior to the ICU admission), the acute disease itself (e.g. sepsis [1]), complications of critical illness (e.g. myocardial ischaemia [2]) or iatrogenic causes (e.g. mechanical ventilation [3]). This may result in physiological changes that require rapid evaluation, treatment and re-assessment following interventions. Echocardiography is a well-suited tool in this environment. It is non-invasive, portable and safe. It also provides immediate results and allows repeated measures at the bedside. However, as anyone who uses this imaging modality can attest, it can be far from simple to perform and interpret a comprehensive echocardiographic study in the critically ill. Adequate scanning is often challenged by mechanical ventilation, the presence of drains and wound dressings, suboptimal patient positioning or recent surgery and hence results can be suboptimal and complex to interpret and integrate in the clinical picture. Furthermore, the complexities of critical illness and the presence of mechanical and pharmacological organ supports complicate the interpretation of potentially limited ultrasound imaging. In this regard insight into such performance and interpretative confounders in the ultrasound imaging of critically ill patients is essential to avoid mistakes.

Large amounts of published data demonstrate the benefit of ultrasound imaging in the critically ill: as a diagnostic tool (detecting right ventricle (RV) dysfunction from mechanical ventilation [4]), providing cardiac function analysis and haemodynamic evaluation [2]. However, similar to learning intensive care medicine, ultrasound cannot be learnt in isolation. An important ‘pearl’ when learning comprehensive echocardiography in the critically ill is to have active mentorship to avoid mistakes, to prevent forming bad habits and to ensure continued education and quality assurance.

Critical care physicians can use comprehensive echocardiography as an extension of examination and further as a sophisticated tool when routine haemodynamics do not offer a clear solution for the specific abnormality or pathology. In this regard integration of history, physical examination, other investigations as well as 2D/Doppler echocardiography findings is needed to determine the most appropriate management for our patients. In this review, we address areas where those learning comprehensive critical care echocardiography can ensure accurate imaging and interpretation of findings. This list is not exhaustive and merely reflects the more common themes.

## Accurate stroke volume assessment

Stroke volume (SV) assessment in the critically ill patient is an essential part of the cardiovascular examination with echocardiography: for haemodynamic assessment, analysis of severity of valvular lesions and cardiac performance [5]. In this regard, accurate imaging is important to get an accurate estimate and we will address some simple methods to obtain this value.

SV is determined by left ventricle outflow tract (LVOT) diameter (assumed to be circular) multiplied by the LVOT velocity time integral (VTI):

$$ \mathrm{SV}=\mathrm{LVO}{\mathrm{T}}_{\mathrm{area}}\times \mathrm{LVO}{\mathrm{T}}_{\mathrm{VTI}} $$

**Fig. 1:**
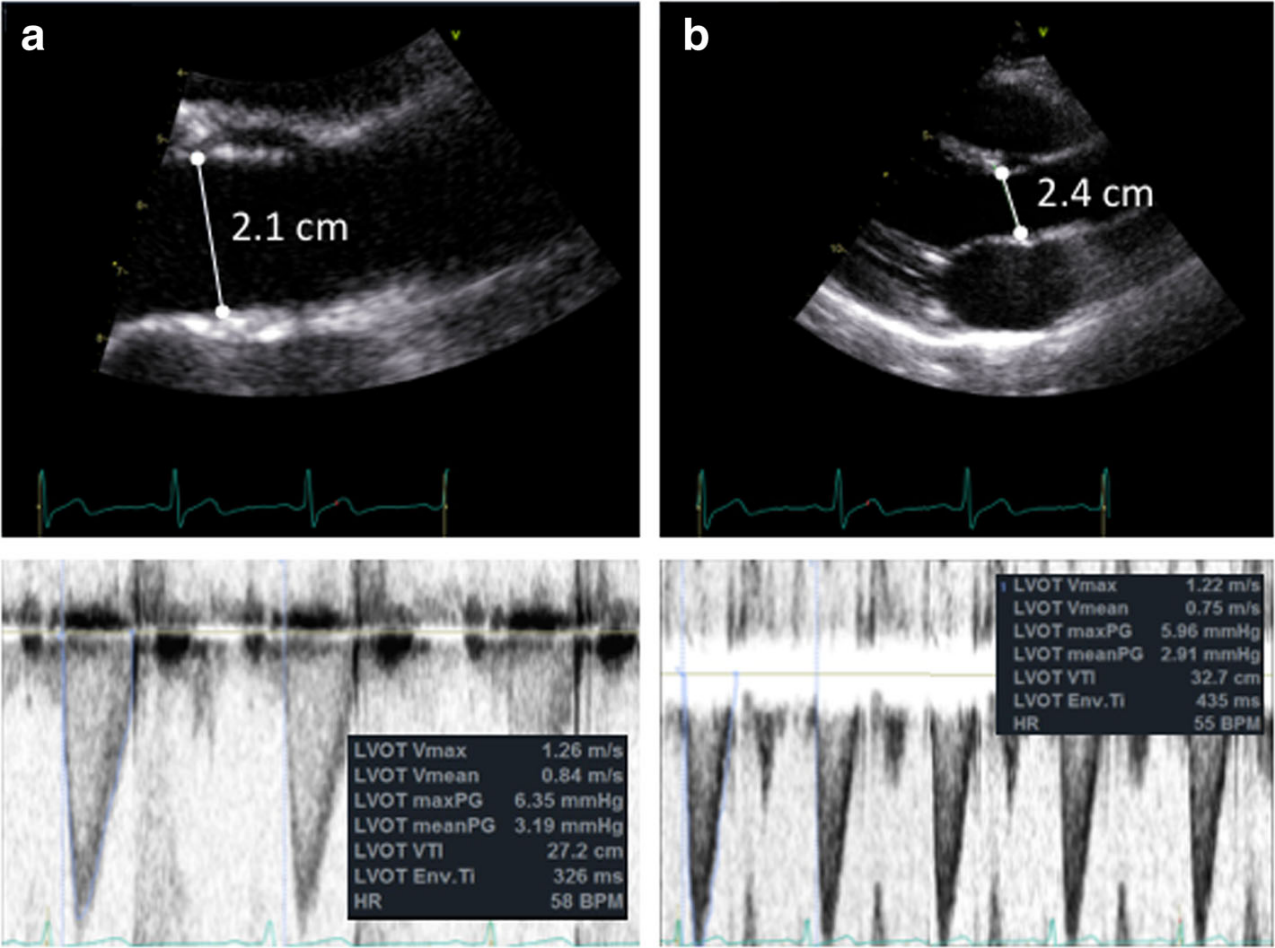
Accurate stroke volume (SV) estimation: SV = LVOT_area_ × LVOT_VTI_. **a** *Accurate SV assessment* = [0.785 × 2.1 cm^2^] × 27.2 cm = 94 ml. LVOT_area_ estimation: use of zoomed in parasternal long-axis view of the aortic valve with LVOT diameter measured at mid-systole at the site of aortic valve cusp entry along with accurate Doppler settings for LVOT VTI assessment using high sweep speed, low wall filters and reduced gain for modal velocity estimation (brightest portion of spectral tracing) as well as seeing the aortic valve closing click. **b** *Inaccurate SV assessment* = [0.785 × 2.4 cm^2^] × 32.7 cm = 148 ml. Potential pitfalls leading to inaccurate SV estimation include mistakes in 2D image acquisition as well as Doppler pitfalls: estimating LVOT_area_ from non-zoomed aortic valve analysis, foreshortened or oblique plane of LVOT interrogation in 2D mode and LVOT spectral Doppler VTI assessment with the sample volume in a wrong position or being too large, with too high gain or too high wall filter settings, low sweep speed and baseline inappropriately low. Note overestimation or underestimation of SV assessment with inappropriate measures

LVOT diameter evaluation should be at the point of entry of aortic valve cusps in a *zoomed* parasternal long-axis view at mid-systole. Correct plane orientation is important in order to identify the largest apparent diameter of LVOT. A foreshortened plane will lead to under-estimation of the stroke volume. Importantly, any inaccuracy in the diameter measurement will be squared (LVOT_area_ = π × LVOT_radius_^2^) increasing the impact of the error on estimation of SV. LVOT VTI assessment is made with a 5–7-mm pulsed wave Doppler (PW Doppler) gate in the LVOT and an aortic valve ‘closing click’ should be seen. This ensures LVOT VTI estimation is at the same site as the LVOT diameter measurement. The PW Doppler settings should be optimised for accurate LVOT VTI estimation: wall filters should be set to low levels and the gain reduced until the brightest (or densest) portion of the spectral tracing is seen, known as the ‘modal velocity’, which represents the velocity of the majority of blood cells (Fig. 1). The outer edge of the modal velocity should be traced for the LVOT VTI [5]. Sweep speed should be reduced to make area assessment more accurate. Two or three cardiac cycles should be averaged for a patient in sinus rhythm and five to seven for a patient in atrial fibrillation. Ideally measures should be made at the same time in the respiratory cycle (e.g. end-expiration for a patient spontaneously breathing) for a patient with significant respiratory variation and particularly when comparing results before and after a treatment is provided.

It is important to note that cardiac output (CO) is the product of SV and heart rate and considering CO without having an estimate of SV can be misleading. For example, a cardiac output of 5 L with a SV of 70 ml may be considered reasonable in a patient on no cardiovascular support with a heart rate of 70 beats per minute (bpm); however, in a patient requiring 25 μg/min of Noradrenaline with a heart rate of 120 bpm and a stroke volume of 40 ml, interpretation would change to ‘a heart under considerable strain’.

**Fig. 2:**
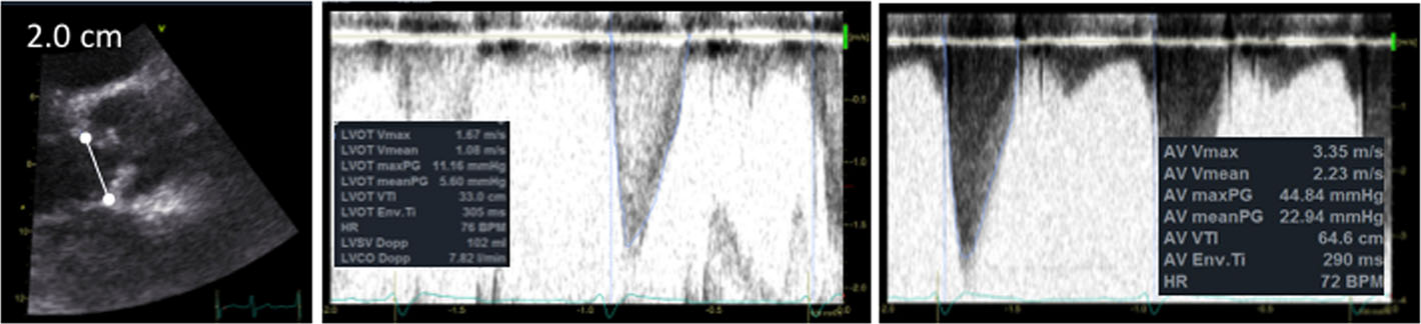
Consideration of stroke volume in valvular lesion severity assessment. In a hyperdynamic state trans-valvular gradients are commonly elevated, as in this example where aortic valve flows could indicate moderate stenosis if considered in isolation. A raised stroke volume and LVOT_VTI_ can help identify falsely elevated flows. In aortic stenosis the VTI ratio between the LVOT and aortic valve can also be of use (Dimensionless Severity Index)

Caution should also be exercised when considering valvular dysfunction in a hyperdynamic state where a sclerotic or mildly stenotic aortic valve can be wrongly assumed to be significantly stenotic if the transvalvular pressure gradient is elevated due to the high flow, as estimated by the continuous wave (CW) Doppler trace, if considered in isolation (Fig. 2).

## Importance of upstream and downstream flows in significant valvular regurgitation

**Fig. 3:**
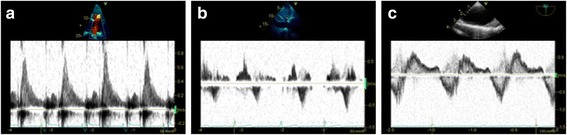
Upstream and downstream flow assessment in moderate or severe valvular lesions. **a** Systolic flow blunting in a pulmonary vein from severe mitral valve regurgitation (upstream). **b** Systolic flow reversal in the hepatic vein from severe tricuspid regurgitation (upstream). **c** Diastolic flow reversal in the descending aorta from severe aortic regurgitation (downstream)

In the critically ill patient significant valvular dysfunction can be perilous if the diagnosis is missed (e.g. severe aortic regurgitation in a patient on an intra-aortic balloon pump). Relying on colour Doppler analysis alone is insufficient for moderate or severe valvular regurgitation due to inaccuracies from gain, scale or eccentrically directed flows and with possible difficulty with imaging. Assessment of flows with continuous wave Doppler is an essential part of valvular lesion severity analysis; however, flows are very much dependent on fluid status as well as cardiac output, i.e. overestimation of regurgitation severity in a fluid overloaded or high cardiac output state. Reviewing the upstream or downstream flows can be a useful technique and relatively simple method of analysis to review if the valvular regurgitation is severe. For example, with severe mitral or tricuspid valve regurgitation there would be systolic flow reversal in the pulmonary or hepatic veins, respectively (upstream flow assessment); with severe aortic valve regurgitation diastolic flow reversal in the proximal aorta is measured from the suprasternal window (downstream flow assessment) (Fig. 3). Specific imaging techniques may help when trying to image smaller vessels (e.g. the pulmonary veins): narrow imaging window width, minimised imaging depth to avoid Doppler aliasing, small PW Doppler gates (~3 mm) all may help achieve an adequate flow trace. In addition, early mitral inflow will have a high peaked E wave velocity (>1.3 m/s) on a PW Doppler profile when there is severe mitral incompetence (assuming ejection fraction > 40%) along with a LVOT VTI < 15 cm. With severe aortic regurgitation, stroke volume estimated by VTI can be abnormally high.

## Performing passive leg raising: do it well

The passive leg raising (PLR) manoeuvre has become one of the reference standards in the ICU to assess fluid-responsiveness at the bedside [6–8]. It has no major risks in appropriately selected patients and the induced effect is reversible. PLR can be used in patients either receiving mechanical ventilation or breathing spontaneously [6, 9]. The PLR should be considered as a replacement to a fluid challenge to avoid unnecessary fluid administration. Cardiac output is assessed before and after this manoeuvre as the assessment of blood pressure alone will not necessarily indicate changes in CO or SV [6]. Cardiac ultrasound is only one of the tools which can be used to estimate response and has been shown to be an accurate method for measuring cardiac output pre- and post-PLR [6].

**Fig. 4:**
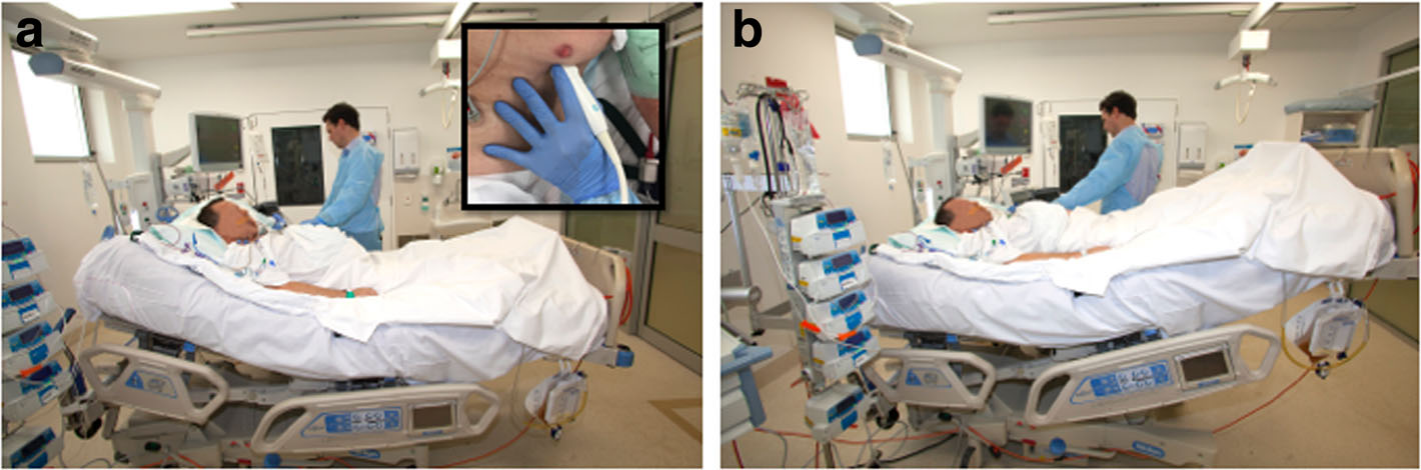
How to perform a passive leg raise correctly: move the patient and the bed from a semi-recumbent position with the head at 30–45° (**a**) to a supine position with the legs raised by 30–45° (**b**) and not by manually raising the patient’s legs alone. NB: ensure that the torso of the patient is horizontal. **a** *Inset*: Pearl = anchor your imaging hand on the patients torso to ensure efficient imaging and the same pulsed Doppler gate position and angle of Doppler interrogation

To be accurate the manoeuvre should follow a strict protocol. If the patient is receiving mandatory mechanical ventilation, tidal volumes of 8 ml/kg should be transiently used. The PLR is performed as shown in Fig. 4 mobilizing the bed and the patient from a semi-recumbent position to a supine position (patient’s torso horizontal) with their legs at 30–45° and not by manually raising the legs. This is because the angle between the trunk and the legs should remain the same to avoid any compression of the femoral vein, which may reduce the venous return to the heart, and to minimize any pain or discomfort with the PLR manoeuvre to avoid adrenergic stress, which may increase heart rate and lead to a misinterpretation of the CO change. Approximately 300 ml of blood is shifted during a PLR when performed properly; however, only the blood from the abdomen will be moved if the PLR is done inadequately through compression of the femoral vein, leading to an underestimation of the fluid requirement [10]. The CO or SV should be assessed 1 minute after the PLR, because at this time the effect seems the highest.

Using echocardiography to assess response to a PLR can be difficult. A few pointers can be used to ensure accurate imaging, which can help with accurate interpretation:

1. Anchor your hand on the patient to keep the same position pre- and post-PLR to ensure efficient imaging and the same pulsed Doppler gate position and angle of Doppler interrogation (Fig. 4a inset)
2. Optimise the LVOT Doppler profile for stroke volume assessment (as per the “Accurate stroke volume assessment” section) with appropriate gain, scale and sweep speed. Assess stroke volume pre-PLR, 1 minute post-PLR and again once supine [11], at the *same time* in the respiratory cycle, using an increase (and subsequent decrease to baseline once supine again) of 15% as a marker of a responder [8].
3. Setting Doppler sweep speed to 3–5 *respiratory* cycles and using PW Doppler in the LVOT or aortic valve, Vmax variations with respiration can be used to assess fluid responsiveness [12] with a cutoff value of 20% (from transoesophageal studies [13]). The heart may move with respiration, however, making the imaging challenging. Observing the clarity of the Doppler profile and ensuring it is similar throughout the screen can indicate if this value can be used or not. In our practice we rely more on stroke volume assessment.

This manoeuvre has several limitations, including high abdominal pressures, which may cause compression of the vena cava, limiting blood shift to the heart and resulting in potential misinterpretation of PLR results [14]. In addition, rapid and accurate measurements of SV are needed (e.g. with Doppler or calibrated pulse contour analysis techniques) and care must be taken not to simply assess response using blood pressure. Indeed, using arterial pulse pressure rather than CO has been criticized due to issues with arterial compliance and pulse wave amplification [11]. Cardiac arrhythmias seem not to be a limitation per se, but five to seven Doppler flow VTI measurements from consecutive beats should be averaged [15].

## Left ventricular ejection fraction—a precarious measurement

Left ventricular (LV) ejection fraction (EF) is often used as an estimate of systolic function and may be a reasonable surrogate in certain patients for adequacy of overall circulatory function. It is a traditional reference value in cardiology, as well as a validated prognostic value in many historic studies. However, in the critically ill, particularly in those with shock, it should be used with caution [16, 17]. LV EF is greatly influenced by mitral regurgitation, LV geometry and loading conditions [18, 19]. If the LV cavity is anatomically small, the LV EF can be misleadingly raised. In these scenarios indexed SV assessments may better reflect haemodynamic status [16] and systolic function can be described by S’ using tissue Doppler imaging (TDI), which is suggested to be less load dependent [20]. In addition, low EF does not necessarily mean low SV and low CO, as can be observed in patients with large end-diastolic LV volume and patients with tachycardia, respectively. Accuracy can be significantly impaired with excessive regional wall motion abnormalities or LV geometric alterations when compared to MRI as a reference standard [21]. In addition, the principles of LV EF evaluation (with Simpson’s biplane for example) do not take into account LV twist and torsion, which are suggested to play a significant role in overall cardiac function and the analysis of which requires advanced, research-based techniques, such as speckle tracking [22]. Bloechlinger et al. [23] investigated the effect of septic shock on LV torsion and found peak torsion and apical rotation were significantly impaired in patients with septic shock vs normals, without a major impairment seen in LVEF. A significant portion of cardiac systolic function is missed by simply assessing ejection fraction alone.

## Left atrial pressure vs diastolic dysfunction: what does it mean in ICU patients?

Impairment of left ventricular diastolic function is frequently seen in ICU patients and should be recognized due to its possible prognostic value [24]. Assessing diastolic function can be a challenge in ICU patients. Recent recommendations [25] propose to assess diastolic function by principally using mitral annulus e’ velocity (using tissue Doppler imaging), mitral inflow E-wave using PW Doppler, the size of the left atrium and tricuspid regurgitation jet. Following these recommendations, normal e’ and normal left atrial size rule out diastolic dysfunction. The authors proposed to grade diastolic dysfunction using different parameters such as E/e’ ratio and E/A ratio of the mitral inflow, which reflect left atrial pressure. This supposes that left atrial pressure is directly related to the degree of diastolic dysfunction.

Unfortunately, in ICU patients left atrial pressure is not entirely related to the diastolic function: a volume-overloaded patient may have a very high left atrial pressure despite a normal left ventricular diastolic function. Conversely, in the presence of hypovolaemia left atrial pressure may be low despite a ‘stiff’ non-compliant left ventricle. Also, patients with septic shock may develop diastolic and systolic dysfunction coexisting with normal left atrial pressure [26]. Hypovolemia may induce diastolic dysfunction and it has been demonstrated that in fluid responders fluid infusion may improve LV relaxation [27]. In ICU patients, evaluation of both diastolic function and left atrial pressure should be interpreted separately. The e’ is potentially the best parameter in ICU patients to assess left ventricular relaxation due to its lesser dependence on the preload [28], although relying on this one parameter alone is likely to be misleading in a great range of pathologies. When using TDI it is important to to ensure accurate imaging: for example by placing the PW Doppler trace at the mitral annulus base and considering that the measurement is angle-dependent. Septal and lateral mitral annular e’ values are usually averaged for the E/e’ ratio. The E/e’ ratio, pulmonary venous flow and E wave deceleration time assessment could each be incrementally useful parameters to assess left atrial pressure [29]. Reference values in the critically ill have not been validated for many echocardiography parameters in the critically ill, especially left atrial pressure. We use E/e’ greater than 14 (averaged value) as predictive for pulmonary artery occlusion pressures greater than 18 mmHg (as a surrogate for raised left atrial pressure) based off data from relatively small single centre studies [30]. Larger, more robust studies are hopefully coming. In addition, the interatrial septum position being fixed and bowed to the right can indicate raised pressures in the left atrium. It is important to take into account an estimation of right-sided pressures as well when assessing the interatrial septum as of course its movement relates to relative pressures of the right vs the left atrium.

## Right ventricle dysfunction: systematic assessment is needed

RV function evaluation in ICU patients receiving mechanical ventilation is challenging [31]. Protective ventilation with high PEEP, low tidal volume and low driving pressures improves outcomes in patients with ARDS [32]. Using a high PEEP in conjunction with ARDS may induce acute cor pulmonale (ACP) with dilation of the RV and pulmonary hypertension. The RV is very sensitive to any changes either in preload or afterload. RV size is commonly assessed from the four chamber apical view. The RV is wrapped around the left ventricle and has a larger cavity volume, a fact frequently misinterpreted due to the 2D nature of traditional ultrasound imaging. From the apical view the RV has a triangle shape and appears to be smaller than the LV (normal ratio between area of the RV and LV < 0.6) [4]. But, if the probe is slightly translated to the right or a rib space too high, the RV could be observed from a position where it looks larger than the LV. To correctly assess the RV size, the probe should be positioned just at the apex of the LV with the interventricular septum parallel to the vertical axis and in the middle of the scanning sector. Rotational optimization of probe position is essential for correct interpretation of the absolute size and size relative to LV, with both RV qualitative and quantitative assessment. Assessment if the LV is dilated is important when determining RV:LV ratio and quantification of RV size can be done independently to determine if there is RV dilation as well.

Paradoxical septal movement can be observed in patients with ACP from the parasternal short axis view, presenting as a D-shape of the left ventricle [4]. Tricuspid annular plane systolic excursion (TAPSE) and TV S’ wave (on TDI) are sensitive to RV systolic function changes, but these parameters are also sensitive to alterations in LV function [33, 34]. Using all available ultrasound windows to interrogate the RV from multiple 2D planes using a combination of M-mode and Doppler measurements will provide incremental information for a comprehensive anatomical and functional evaluation of this complex structure. Dynamic evaluation with variable PEEP may prove invaluable for selecting the most appropriate ventilator settings and optimizing heart–lung interactions in a highly individualized patient approach.

## Recognise all the information provided in a Doppler profile

**Fig. 5:**
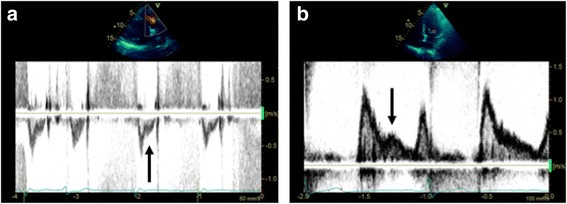
Additional information provided by Doppler flow pattern analysis. **a** Flying ‘W’ sign with pulsed wave Doppler analysis of right ventricle outflow tract: a highly specific sign of pulmonary hypertension. **b** L-wave on mitral valve inflow: seen in relatively bradycardic patients with normal hearts or in a pathological setting in those with elevated left ventricle preload and moderate diastolic dysfunction

The PW Doppler profile is commonly used for estimating haemodynamics and pressure gradients, but the pattern of flow velocities can also be interpreted. Most of these findings were described many years ago, often through M-mode analysis, long before the Doppler technology became widely available. Analysis of Doppler flow patterns in the RV outflow tract may provide indirect information regarding pulmonary haemodynamics. In significant pulmonary hypertension reduced acceleration time and premature closure of the pulmonary valve may occur, resulting in a mid-systolic notch, known as the “flying ‘W’ sign” (Fig. 5a) [35]. The notch may be caused by reduced pulmonary artery compliance or increased pulmonary artery impedance and is highly specific for pulmonary hypertension [36].

The mitral ‘L-wave’ is a relatively common, useful and often ignored finding [37] where mid-diastolic flow is seen across the mitral valve attributed to pulmonary vein flow continuing through the left atrium into the LV after early rapid filling (Fig. 5b). The L wave may be seen in relatively bradycardic patients with normal hearts, but is typically seen in a pathological setting with increased LV stiffness or delayed active relaxation and is suggestive of elevated LV preload and moderate diastolic dysfunction. It is prognostic of future adverse events in patients with heart failure [38]; however, it has not been evaluated closely in critically ill patients as yet.

## Left ventricular obstruction: don’t miss it

**Fig. 6:**
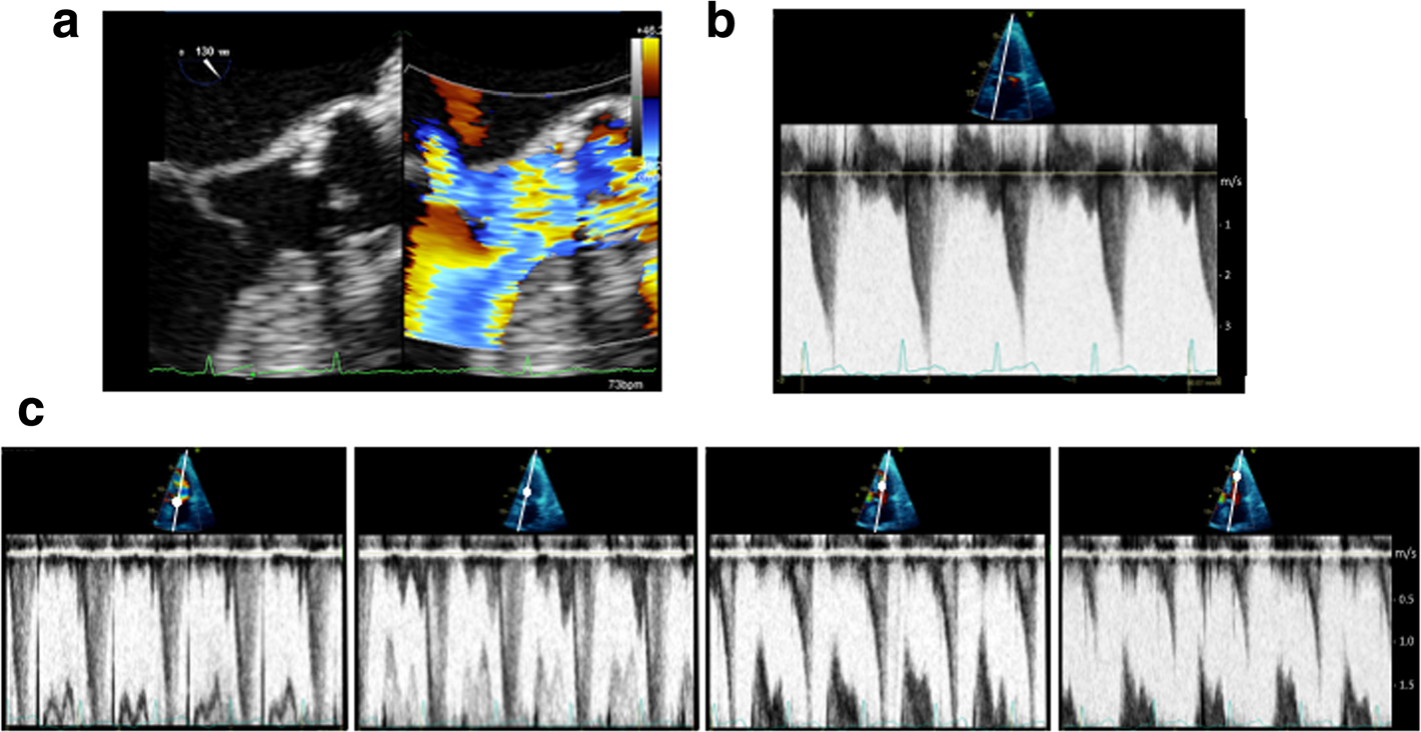
Left ventricular cavity obstruction. Obstruction of the left ventricular cavity can be due to anatomical factors and/or precipitated by LV cavity obstruction from hypovolaemia, excessive inotropic agents, predisposing anatomical abnormalities, etc., **a** systolic anterior motion of theanteiror mitral valve leafelet is frequently seen, often with resulting mitral regurgitation. Accurate imaging to determine location of the obstruction is required. **b** Continuous wave Doppler can identify an obstruction through recognition of the classic ‘dagger’ shaped curve with its peak in late systole. **c** Pulsed wave Doppler can identify the point of restriction by sequentially moving the gate from the left ventricle outflow tract to the apex looking for the point of maximal flow (note that aliasing is frequently seen where the maximum gradient is too high for the pulsed wave Doppler scale)

Intra-cavity obstruction of the LV was initially described in patients with hypertrophic cardiomyopathy and asymmetric LV hypertrophy [39, 40]. However, LV obstruction can also occur in ICU patients where significantly reduced afterload, generalized hypertrophy and hypovolaemia can lead to subvalvuar obstruction and/or mid-cavity obstruction. For example, it may occur in up to 22% of patients with septic shock [41] and is associated with a high mortality rate. Obstruction is usually due to a combination of anatomical abnormalities and precipitating factors [42, 43]. Anatomical abnormalities that can contribute to LVOT obstruction include LV hypertrophy, sigmoid septum, regional wall motion abnormalities, apical ballooning, misplacement of mitral valve prosthesis or after mitral valve repair [44]. Precipitating factors includes factors which increase LV contractility or decrease preload or afterload. For example, hypovolaemia, sepsis and inotropic agents may precipitate obstruction in the presence of (or even sometimes the absence of) anatomical abnormalities. When present, LV obstruction can be found either at the LVOT level or at the mid-ventricle level by using spectral Doppler modalities. The tell-tale signs include a very small LV cavity, which can give the impression of pseudohypertrophic walls [45]. Zoomed views, with high frame rates, to assess the LVOT may frequently reveal the presence of systolic anterior motion (SAM) of the anterior mitral valve leaflet (Fig. 6a). In these circumstances LV Doppler assessment should be done with caution. PW Doppler should be used and the sample volume moved slowly from the apex to the base and into the LVOT to check for any dagger-shaped flow. Simultaneous multiple points of intra-cavity obstruction may coexist. Colour Doppler may be very useful to help isolate the point of restriction and CW Doppler should be used to check for maximal gradients (Fig. 6b, c). LV opacification with ultrasound contrast can be very helpful in identifying patients with eccentric and apical LV hypertrophy, non-compaction and intracavity masses responsible for fixed or dynamic obstruction. In ICU patients obstruction is most commonly associated with hypovolemia (low afterload can contribute to this), which should be corrected. Infusion of positive chronotropic and inotropic agents in patients after aortic valvular replacement is a frequent precipitating factor and should be stopped or minimized, particularly when the absence of LV systolic failure is echocardiographically demonstrated. Other treatment considerations include using beta blockers in patients with LVOT obstruction and reducing pacemaker output rates [46].

## Use of non-standard imaging windows for Doppler angle optimisation

Clinically important information can be missed by using the conventional echocardiography views only. Non-standard imaging windows may be important, particularly to optimize Doppler angles.

1. Supra sternal view. The probe is positioned behind the suprasternal notch and allows visualization of the great vessels arising from the heart. This view may be considered a part of a standard study in cardiology but is often forgotten in the ICU population. The aortic arch can be examined and aortic flow can be assessed. Aortic arch dissection can be seen, diastolic flow reversal assessed to help quantify aortic regurgitation [47] or potentially a better Doppler alignment with the flow in aortic stenosis obtained. In addition, CO assessment has been validated from this view [48]. Assessment of aortic arch flow can be important for patients on VA ECMO, in particular patients with peripheral ECMO cannulas, in order to ascertain the prevalence of aortic arch flow from native pulsatile cardiac output vs continuous VA ECMO arterial return flow.
2. Apical RV-centric views. The probe is moved medially from the apical position (Fig. 7), potentially providing better visualization of the RV free wall and an improved angle for tricuspid regurgitation analysis.

**Fig. 7:**
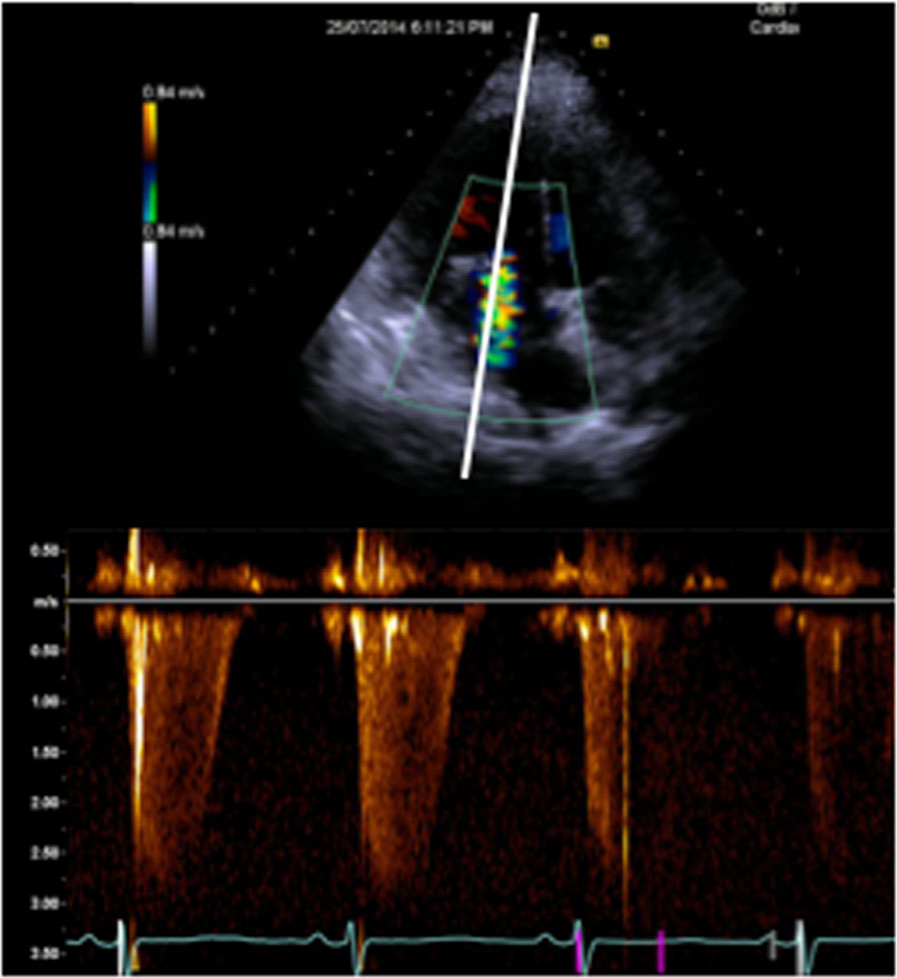
Utility of using non-standard imaging windows. Apical right ventricle-centric views (probe positioned more medially than standard apical view): enables better Doppler angle for tricuspid regurgitation assessment
3. With dilated cardiomyopathy, blood flow from the left atrium into the LV can become increasingly eccentric and directed towards the lateral wall. Using colour Doppler to view direction of blood flow into the LV and placing the probe more laterally can help ensure an accurate Doppler angle.

## Imaging the apex

**Fig. 8:**
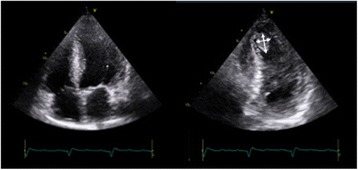
Imaging the apex: off-axis imaging. Standard apical views can miss an anteriorly placed thrombus. If there is a degree of suspicion (e.g. akinetic apical segments), off-axis imaging (i.e. tilting imaging plane or imaging more medially) can be used to identify an apical thrombus (indicated by *arrows*)

The apex of the heart can be particularly challenging to assess. In patients with apical akinesia (e.g. left anterior descending coronary artery infarct, Takotsubo’s cardiomyopathy etc.) the presence of thrombus needs to be excluded. Reducing imaging depth, focus position and increasing frequency can all help visualize the apex from standard apical views, but by moving the probe laterally or medially and angling back towards the apex the entire structure may be visualized (Fig. 8). If available, LV opacification with ultrasound contrast is a valuable technique for assessment of the endocardial border, exclusion of intracardiac masses (especially LV apical thrombus), enhancement of Doppler signals and perhaps regional myocardial perfusion [2]. In addition, when pathology is being searched for at the apex (e.g. ischaemic ventricular septal defects), consider fanning through the entire structure, using off-axis imaging as well as recording images in multiple planes.

## Inferior vena cava assessment

Inferior vena cava (IVC) assessment is a standard part of most comprehensive studies and is used most commonly in assessing fluid responsiveness and in determining right atrial pressure. The evidence for this, however, is often conflicting and shows weak correlation [49] and studies are difficult to compare or combine as they have different reference standards, measure IVC at different times in the cardiac and respiratory cycles, with different views and at different sites or use different modalities (e.g. M-mode vs 2D) [50].

In our practice we find the IVC is useful for right atrial pressure (RAP) estimation but is only reliable in spontaneously breathing patients [51] and is appropriate in categorising RAP into low (0–5 mmHg), normal (6–10 mmHg) or high (greater than 11 mmHg). Additional information such as right atrial size, hepatic flow, tricuspid regurgitation and right heart function assessment is useful to consider in addition to IVC size [52].

In assessment of fluid responsiveness, IVC size and response to respiration is fraught with multiple confounders which need to be considered when making interpretations. RV dysfunction and tricuspid regurgitation can impede venous return and result in a plethoric IVC which shows no correlation with fluid responsiveness [50]. Increased abdominal pressure, large intrathoracic pressure swings (e.g. asthma), pressure-supported breathing and high PEEP can also impact on analysis. In our practice IVC assessment in fluid responsiveness is clinically useful only at extremes and if the IVC is plethoric then all potential confounders mentioned above must be considered.

From a technical perspective, measuring the IVC too close to the right atrium, hepatic vein or diaphragm should be avoided and whichever methodology is being used to measure the IVC should be used every time. 2D assessment is likely better than M-mode as it avoids translational error [53].

## Conclusions

Comprehensive critical care echocardiography is a useful, rapid and non-invasive method to both diagnose pathology and monitor treatment response in the critically ill. Although growing dramatically in use around the world, it is not without its limitations and user dependency is a significant challenge. The two primary objectives of accurate imaging and accurate interpretation are vital and should underpin future growth in the speciality. Good knowledge and attention to detail are vital to avoid mistakes.

## Abbreviations

ACP: Acute cor pulmonale
ARDS: Acute respiratory distress syndrome
CO: Cardiac output
CW: Continuous wave
EF: Ejection fraction
ICU: Intensive Care Unit
IVC: Inferior vena cava
LV: Left ventricle
LVOT: Left ventricle outflow tract
PEEP: Positive end-expiratory pressure
PLR: Passive leg raise
PW: Pulsed wave
RV: Right ventricle
SAM: Systolic anterior motion
SV: Stroke volume
TDI: Tissue Doppler imaging
VTI: Velocity time integral
